# Detecting knee osteoarthritis and its discriminating parameters using random forests

**DOI:** 10.1016/j.medengphy.2017.02.004

**Published:** 2017-05

**Authors:** Margarita Kotti, Lynsey D. Duffell, Aldo A. Faisal, Alison H. McGregor

**Affiliations:** a Musculoskeletal (MSK) Laboratory, Division of Surgery, Department of Surgery and Cancer, Faculty of Medicine, Imperial College London, Charing Cross Hospital, London W6 8RF, UK; b Brain Behaviour Laboratory, Department of Bioengineering, Imperial College London, SW7 2AZ London, UK; c Department of Medical Physics and Biomedical Engineering, University College London, Gower Street, WC1E 6BT London, UK; d Department of Computing, Imperial College London, SW7 2AZ London, UK; e MRC Clinical Sciences Centre, Faculty of Medicine, Imperial College London, Hammersmith Hospital Campus, London, UK

**Keywords:** Knee osteoarthritis, Machine learning, Random forests, Ground reaction forces, Osteoarthritis, OA, Ground reaction forces, GRFs

## Abstract

This paper tackles the problem of automatic detection of knee osteoarthritis. A computer system is built that takes as input the body kinetics and produces as output not only an estimation of presence of the knee osteoarthritis, as previously done in the literature, but also the most discriminating parameters along with a set of rules on how this decision was reached. This fills the gap of interpretability between the medical and the engineering approaches. We collected locomotion data from 47 subjects with knee osteoarthritis and 47 healthy subjects. Osteoarthritis subjects were recruited from hospital clinics and GP surgeries, and age and sex matched healthy subjects from the local community. Subjects walked on a walkway equipped with two force plates with piezoelectric 3-component force sensors. Parameters of the vertical, anterior–posterior, and medio-lateral ground reaction forces, such as mean value, push-off time, and slope, were extracted. Then random forest regressors map those parameters via rule induction to the degree of knee osteoarthritis. To boost generalisation ability, a subject-independent protocol is employed. The 5-fold cross-validated accuracy is 72.61% ± 4.24%. We show that with 3 steps or less a reliable clinical measure can be extracted in a rule-based approach when the dataset is analysed appropriately.

## Highlights

• An algorithmic method that detects knee osteoarthritis.
• Machine learning, specifically random forests, is applied on ground reaction forces.
• Discriminating parameters of knee osteoarthritis are automatically detected.
• Parameters have a clinical interpretation and are in line with medical literature.
• The proposed approach is subject-independent.

## 1 Introduction

Osteoarthritis (OA) rates are rising, in part a reflection of our growing ageing population. Currently OA is the second leading cause of disability [1], and one of the most common forms of arthritis worldwide, accounting for 83% of the total OA burden [2]. The global prevalence of knee OA is over 250 million people [2], according to Vos et al*.* Currently diagnosis of OA is based upon patient-reported symptoms and X-rays. The alternative is MRI but this is associated with high cost and is rarely used until symptoms progress and patients are referred for specialist surgical opinion. Thus effective management and early identification of knee OA is a key health issue and is of interest to the population at large as well as a range of clinicians and health service managers. The method presented here represents an effective solution with significantly lower costs compared to MRIs and ultimately aims to be used as a part of standard clinical assessment for the general population, in contrary to imaging that requires severe symptoms to be present. For all the aforementioned reasons, our vision and our long-term motivation is to develop a diagnostic tool for automatic detection of early markers of knee OA that does not act as a black box for the clinical personnel, as is the common case today.

In this paper, we propose a computer system that uses computational methods from the area of machine learning to estimate the degree of knee OA. This approach overcomes limitations of previous methods, such as Astephen et al*.* [4], Federolf et al*.* [6], Beynon et al*.* [8], Deluzio and Astephen [9], and Mezghani et al*.* [11], in the sense that it (i) automatically estimates the degree of knee OA by recognising patterns that are more discriminating of knee OA; (ii) discriminates the most important parameters for reaching its decision; and (iii) produces a set of rules that have a clear clinical rationale. Machine learning concerns the construction of computer systems that are able to learn from data. Such approaches have recently been adopted by the biomechanical field with great effect. The common trend in biomechanics research is to consider individual parameters such as flexion moment peak value, or rotation moment, as done by Kaufman et al*.* [3] and then statistically test if there are significant differences in each parameter between the patients and normal subjects. However, machine learning looks at the complexity of the data as a whole [4], overcoming limitations that arise from hypothesis testing using individual parameters, thereby losing the richness and complexity of the data. For example, machine learning can be used to interpret electromyographic, kinematic and kinetic data from the knee, hip and ankle joints during gait and has been shown to be able to separate healthy patients, mild, and severe knee OA according to Haber et al*.* [5]. Federolf et al. [6] identified systematic differences between healthy and medial knee-osteoarthritic gait using principal component analysis. In this study we analyse parameters of ground reaction forces (GRFs) to estimate using an objective scale the degree of knee OA and to extract parameters that differentiate more effectively between normal and knee OA subjects. To the best of the authors’ knowledge, this is the first study on detecting knee OA via analysing the GRFs using random forests. We believe that a purely data-driven approach yields objective measures and patterns useful for both biological and clinical advancement as suggested by Faisal et al*.* [7]. Emphasis is given on detecting parameters with physical meaning and in inducting rules that remain fully interpretable even to non-data analysis experts. The guidance rules may be adopted in a routine clinical visit to provide support to healthcare professionals during decision-making. Our final aim is to derive a software tool that can be used either to assist the physician when diagnosing new patients or to train students to diagnose patients.

Previous biomedical studies by Beynon et al*.* [8], Deluzio and Astephen [9], Moustakidis et al*.* [10], and Mezghani et al*.* [11] have discriminated between subjects with knee OA versus normal subjects, as detailed below. For example Beynon et al*.* [8] explored the use of sagittal/frontal/transverse plane range of motion and the peak vertical ground reaction force during the stance phase of gait and cadence. They were able to discriminate knee OA subjects (total 30 subjects, 15 with knee OA, 6 gait cycles per subject) using the Dempster-Shafer theory of evidence. Depending on whether the proposed method's heuristic values are computed by descriptive statistics or provided by an expert, the system had a performance of 90% or 96.7% respectively. In another study by Deluzio and Astephen [9] 50 patients with end-state knee OA and 63 control subjects performed five walking trials. Knee flexion angle, flexion moment, and adduction moment were classified using linear discriminant analysis after principal component analysis, achieving a 93% correct classification. More recently, GRFs have been studied. Wavelet analysis by Moustakidis et al*.* [10] has shown that a reduction in peak anterior–posterior ground reaction forces during the stance phase occurs in knee OA subjects (12 healthy, 24 with knee OA). They were grouped in no, moderate, and severe OA categories with a 93.4% performance. A second study by Mezghani et al*.* [11] calculated the coefficients of a polynomial expansion and the coefficients of wavelet decomposition for 16 healthy and 26 tibiofemoral knee OA subjects. A nearest neighbour classifier achieved accuracies ranging from 67% to 91%, depending on the set of parameters.

The main objective of this work is to give emphasis to clinicians’ rationale. That is the reason why we refrain from abstract mathematical approaches such as wavelet packet decomposition as done by Moustakidis et al. [10], as they lack a direct physical interpretation. Moreover, we consider all the trials provided by each subject, rather than averaging across trials in order to calculate the mean GRFs, as is the case of Mezghani et al*.* [11]. Averaging disregards the intra-subject variability. While previous work focussed on predicting discrete outcomes, our approach provides a continuous number between 0 and 2, since we felt that clinicians would value a continuous output, rather than a yes/no answer, whilst at the same time reflecting the progressive degenerative nature of osteoarthritis. Very few previous studies provide an alternative to discrete predictions. Beynon et al*.* [8] provided a level of belief that a subject has knee OA or is normal and the associated level of uncertainty. Finally, our approach does not adopt any ad hoc heuristics, like the one proposed by Beynon et al*.* [8].

It is worth mentioning that the focus of machine learning does not have to be knee OA prediction. For example, the authors Favre et al*.* [12] applied neural networks to predict knee adduction moment during walking based on ground reaction force and anthropometric measurements, whereas Begg and Kamruzzaman [13] applied support vector machines to discriminate young from elderly subjects exploiting kinetic and kinematic parameters, and Muniz et al*.* [14] evaluated Parkinson disease exploiting GRFs. Accordingly, the proposed system here is tackling the problem of estimating the presence of knee OA via a rule based approach that concurrently estimates the most discriminating features of the pathology. However, it could also be utilised to analyse additional musculoskeletal diseases, like back pain, given the respective kinetic parameters for its re-training.

## 2 Materials and methods

In this study, subjects diagnosed with OA were recruited, along with gender and age matched control subjects. We collected locomotion data from 47 subjects with knee osteoarthritis and 47 healthy subjects. The mean value and the standard deviation between normal and knee OA subjects of the age, height, weight, and sex for the 47 controls and the 47 knee OA subjects are depicted in Table 1. Ethical approval for this study was obtained from the South West London Research Ethics Committee and written informed consent was obtained from all participants. Control subjects were recruited from local university and hospital staff and students. OA subjects were recruited from hospital clinics and local General Practitioner (GP) clinics. Presence of OA was confirmed from medical reports and clinical examination by their practitioner. Subjects were excluded from the study if they reported any neurological or musculoskeletal condition other than knee OA, rheumatoid or other systemic inflammatory arthritis, morbid obesity (Body Mass Index >35 kg/m^2^) or had undergone previous surgical treatment for knee OA.

Subjects were asked to walk at their self-selected walking speed along a 6 m walkway embedded with two force plates (Kistler Type 9286B, Kistler Instrumente AG, Winterthur, Switzerland). Kistler Type 9286B force plate exploits piezoelectric 3-component force sensors. It has 4 measuring elements, one at each corner of the 600 mm × 400 mm force plate. It has a rigidity of ≈12 N/μm for the *x* and the *y* axes and of ≈8 N/μm for the *z* axis. The linearity for all GRFs is <±0.2% FSO and the respective hysteresis equals <0,3% FSO. Measuring range is −2.5 to 2.5 kN for GRFX and GRFY, whereas the respective range for GRFZ is 0 to 10 kN. Each subject was barefoot and unaware of the force plates embedded in the walkway. Each subject was asked to walk along the walkway three times. Trials with no clean force plate strike were excluded. A maximum of three trials were recorded for the left and right foot. The signals from the force plates were recorded using an analogue signal data acquisition card provided with the Vicon system (Vicon Motion Systems Ltd, Oxford, UK) and the Vicon Nexus software at a sampling rate of 1000 Hz.

GRF data was extracted, normalised to the subject's body weight (N/kg), to reduce inter-subject variability due to weight, and time-normalised to the entire gait cycle using linear interpolation. Next, statistical parameters were extracted for each axis. A list of those that are common among the three axes is available in Table 2.

**Fig. 1:**
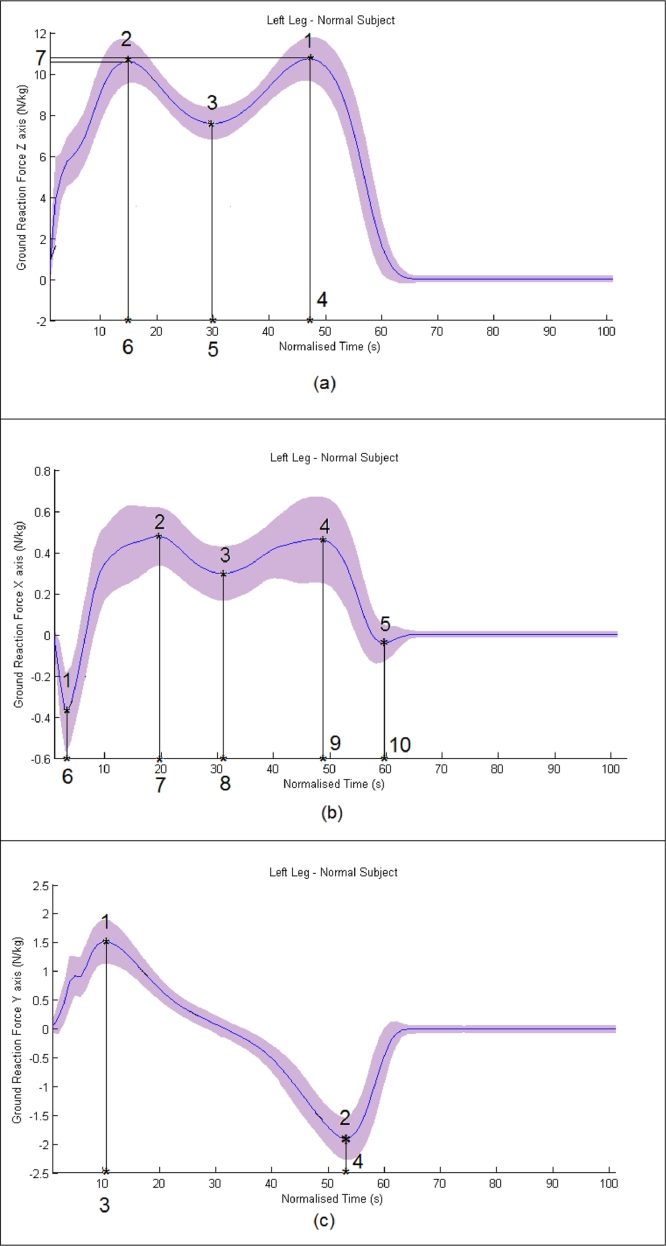
(a) GRF-Z for the left foot of control subjects. The blue curve corresponds to the mean GRF-Z curve, whereas the purple shaded region indicates the precision of plus minus one standard deviation. Parameter 1 is the second peak, parameter 2 is the first peak, parameter 3 is the minimum of the middle stance value, Parameters 4, 5, and 6 are the respective time stamps of the aforementioned extremes. Parameter 7 is the difference of parameter 2 minus parameter 1. Two ratios are also calculated: the first is the ratio of parameter 1 over parameter 3 and the second ration is that of parameter 2 over parameter 3. (b) GRF-X for the left foot of control subjects. The blue curve corresponds to the mean GRF-X curve, whereas the purple shaded region indicates the precision of plus minus one standard deviation. Parameter 1 the minimum during loading response, parameter 2 is the maximum of middle stance, parameter 3 is the minimum of the joint middle stance, parameter 4 the maximum of terminal stance, and parameter 5 is the minimum of the terminal stance. Parameters 7, 8, 9, and 10 are the respective time stamps of the aforementioned extremes. (c) GRF-Y for the left foot of control subjects. The blue curve corresponds to the mean GRF-Y curve, whereas the purple shaded region indicates the precision of plus minus one standard deviation. Parameter 1 is the peak and parameter 2 is the minimum value of the stance phase. Parameters 3 and 4 are the respective time stamps of the aforementioned extremes.

Additionally, axis-specific parameters are extracted. For the Z-axis the first peak, second peak, and minimum of the mid stance values were calculated along with the time stamps of those events. Furthermore, the differences between the values recorded from each leg were calculated. Also, the difference between the first peak and the second peak was calculated. Finally, two ratios were calculated: the ratio of the 1st peak value over the minimum value during mid-stance and the ratio of the 2nd peak value over the minimum value during middle stance. The difference between the two aforementioned ratios was also calculated. The aforementioned parameters are graphically depicted in Fig. 1(a). For the X-axis, the minimum during loading response, the maximum of mid stance, the maximum of terminal stance, and the minimum of mid stance and terminal stance were considered. Once again the time stamps of those values are taken into account. Those parameters can be seen in Fig. 1(b). Accordingly, for the Y-axis, the maximum and the minimum values are taken into account along with the respective time stamps, as is demonstrated in Fig. 1(c). For each GRF several slopes are defined between two successive extremes. The asterisks in Fig. 1 denote the extremes. Additional extremes exist at the beginning and the end of the stance phase. For example, the GRF of the Z-axis has one slope defined from the beginning of the gait cycle to the 1st peak. This protocol also applies for the GRFs for X and Y-axes. More specifically, 6 slopes were calculated for the GRF over X-axis and 3 for the Y-axis.

The advantage of this parameter extraction method is that these parameters bear a physical meaning. The more abrupt the slopes, the quicker that phase occurred relative to the gait cycle. Interquartile range, as well as median is more robust to outliers than the mean. Spearman correlation between left and right legs estimates the strength of the associations of the gait patterns, since knee OA sufferers tend to overload one leg at the expense of the other, as evidenced in Duffell et al*.* in [15]. It is normal to assume that even if just one knee suffers from OA the patterns of the other knee may be altered. GRF-Z demonstrates two peaks, the first reflects weight transfer from the heel to the mid-foot and the second one is related to the ball of the foot for push-off, as mentioned by Alaqtash et al. in [16]. Also, there is a minimum during the stance phase. These three extremes define an M-shape. The ratios that are calculated for GRF-Z are estimations of its M shape, as explained by Alaqtash et al. [16] and Takahashi et al*.* [17].

With respect to the ensemble, random forests take the input parameters, traverse them with every tree in the forest, and then average the responses over all the trees. Specifically, each tree considers a different random subset of the parameters. By this procedure, called *bagging*, different trees have different training parameter sets. Moreover, for each tree node a subset of the training parameter set is considered. The final regression value is obtained by averaging the regression values of the random trees, as proposed by Breiman [18]. Random forests need no cross-validation according to Breiman [18], this procedure happens inherently by selecting a subset of parameters for every tree and node. Random forests perform parameter selection automatically. If a feature is of poor discriminating ability it will not appear in any node of the trees comprising the forest. Accordingly, if a feature is highly informative it will not only appear in several trees, but will also have a tendency to appear to nodes that are more close to the root, as explained by Chen and Ishwaran [19]. Here, a Matlab (Matlab 2012b, The MathWorks Inc., Natick, MA, 2012) implementation of random forests is utilised. To select the most informative parameters, we compute the increase in prediction error if the values of that parameter are permuted across the out-of-bag observations. Out-of-bag observations are those that are left out during the construction of each tree. Since we construct each tree using a different bootstrap sample from the original data that includes the two thirds of the cases, the remaining one-third is left out, constituting the out-of-bag observations. The increase in the prediction error if the values of that parameter are permuted across the out-of-bag observations is computed for every tree, then averaged over the entire ensemble and divided by the standard deviation over the entire ensemble. For this work, we report the 3 most informative parameters per axis. We used half of the subjects’ trials for creating the random forest and the other half for testing in a subject independent manner. This means that the two sets (training and testing) are disjoint, to ensure good generalisation ability. The output of the method is a regression value ranging from 0 to 2, in order to support clinicians with their decisions. We focus on regression instead of classification since we believe that for a clinician it is more useful to obtain a continuous value rather than whether the subject does or does not have knee OA. Also, OA is a degrading disease. The closer this value is to 0 the more probable the subject under consideration is a healthy one, i.e. exhibits no knee OA. A value of 2 equates to both knees suffering from severe OA. In all, a patient may be considered to exhibit no OA if the system calculates a value less than 0.5.

The performance of our system was assessed in a subject-independent manner, i.e. by completely separating the training data (used to create the random forests) from the test data (used to assess the performance). Specifically, we trained each regression forest on half the number of trials, which corresponded to 48 subjects. Half of them were suffering from knee OA and the remainder were healthy. We then tested the efficiency of the proposed approach on the remaining trials carried out by 46 subjects. This means that the testing data has never been seen before by the regression forest, rendering the system robust to generalisation and handing of new, unknown subjects. The experimental protocol is subject-independent. If a subject's trial is included in the training set, then all the trials of this subject are part of the training set and are not used in the test set. This way, the system is able to handle efficiently an unknown subject; is robust; and permits generalization. Since each subject provides up to 3 gait cycles, the output is averaged over the gait cycles, so as to have one final regression value per subject per GRF plane.

## 3 Results

**Fig. 2:**
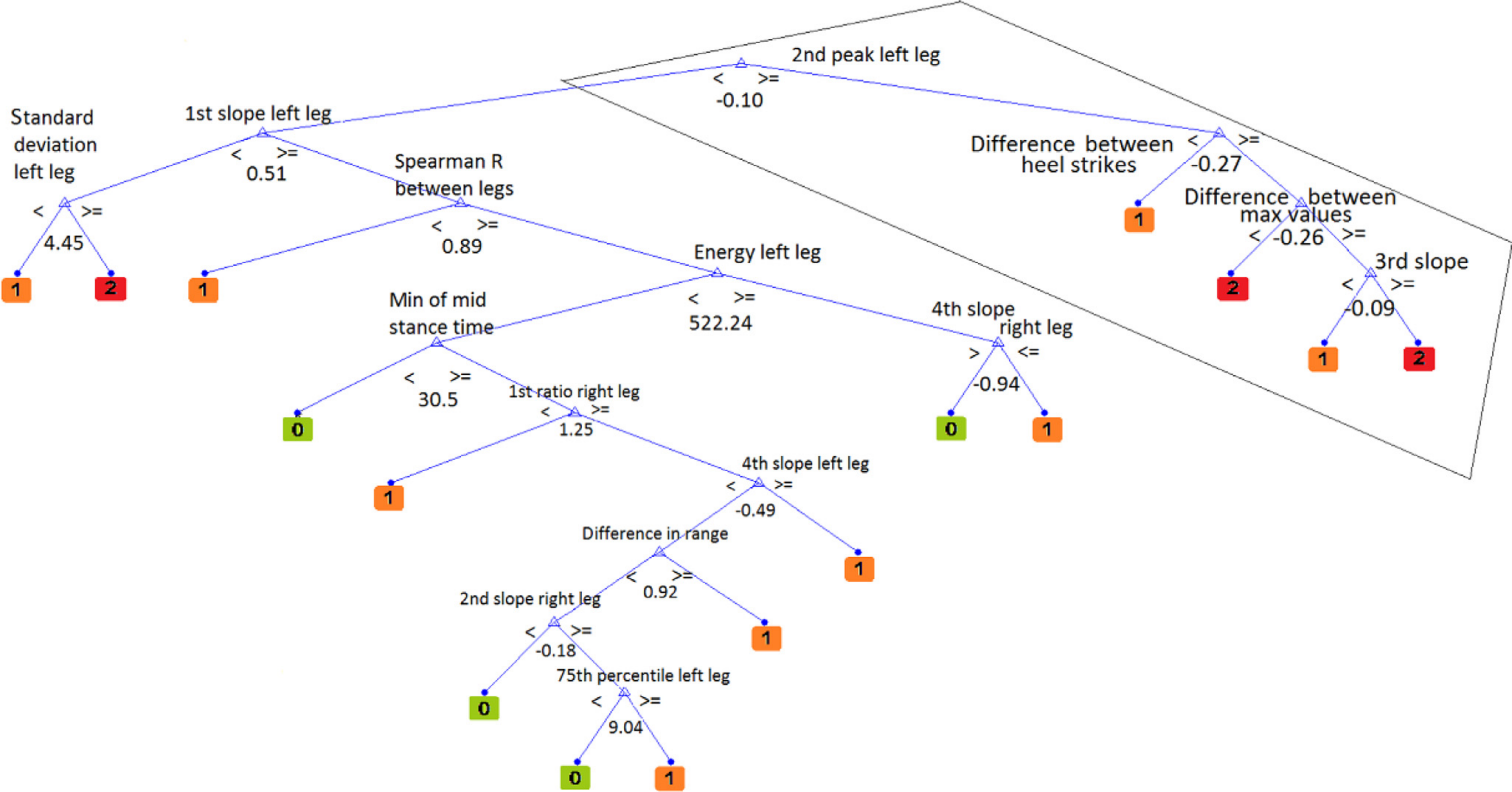
One of the regression trees comprising the random forest for the GRF over *Z*-axis. The regression tree is built using a random subset of the parameters extracted for GRF-Z. The highlighted area in the trapezoid is demonstrated in more detail in Fig. 3, so as to give a more detailed idea of the rule induction. A value of 0 indicates a training subject that has no knee OA, of 1 that is clinically diagnosed with OA in one knee and with 2 suffering OA in both knees.

**Fig. 3:**
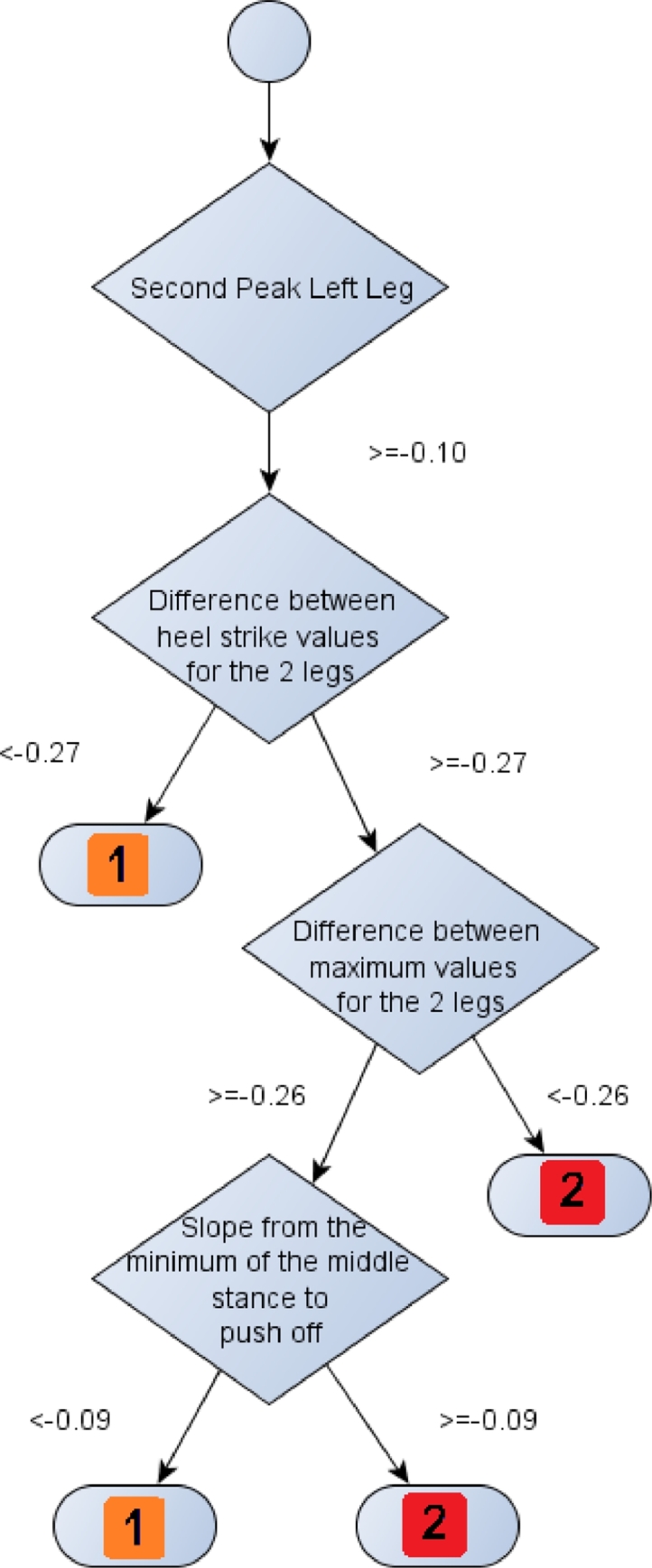
A part of the highlighted branch of the regression tree depicted in Fig. 2. The branch, which is given by means of a flowchart, follows a rule induction approach to reach conclusions on the degree of knee OA based on binary decisions of the parameters extracted. A value of 0 indicates a training subject that has no knee OA, of 1 that is clinically diagnosed with OA in one knee and with 2 suffering OA in both knees.

For visualisation purposes, one tree out of the ten that comprise the random forest is depicted. Accordingly, a tree that traverses GRF-Z is depicted in Fig. 2 and the respective trees for GRF-X and GRF-Y are depicted in electronic supplementary material, Figs. S1 and S2, respectively. A close up of one branch of the tree demonstrated in Fig. 2 can be seen in Fig. 3. The aim of Fig. 3 is to focus solely on one branch of the tree, so as to provide a better insight to the nature of the binary rule induction implemented by trees.

**Fig. 4:**
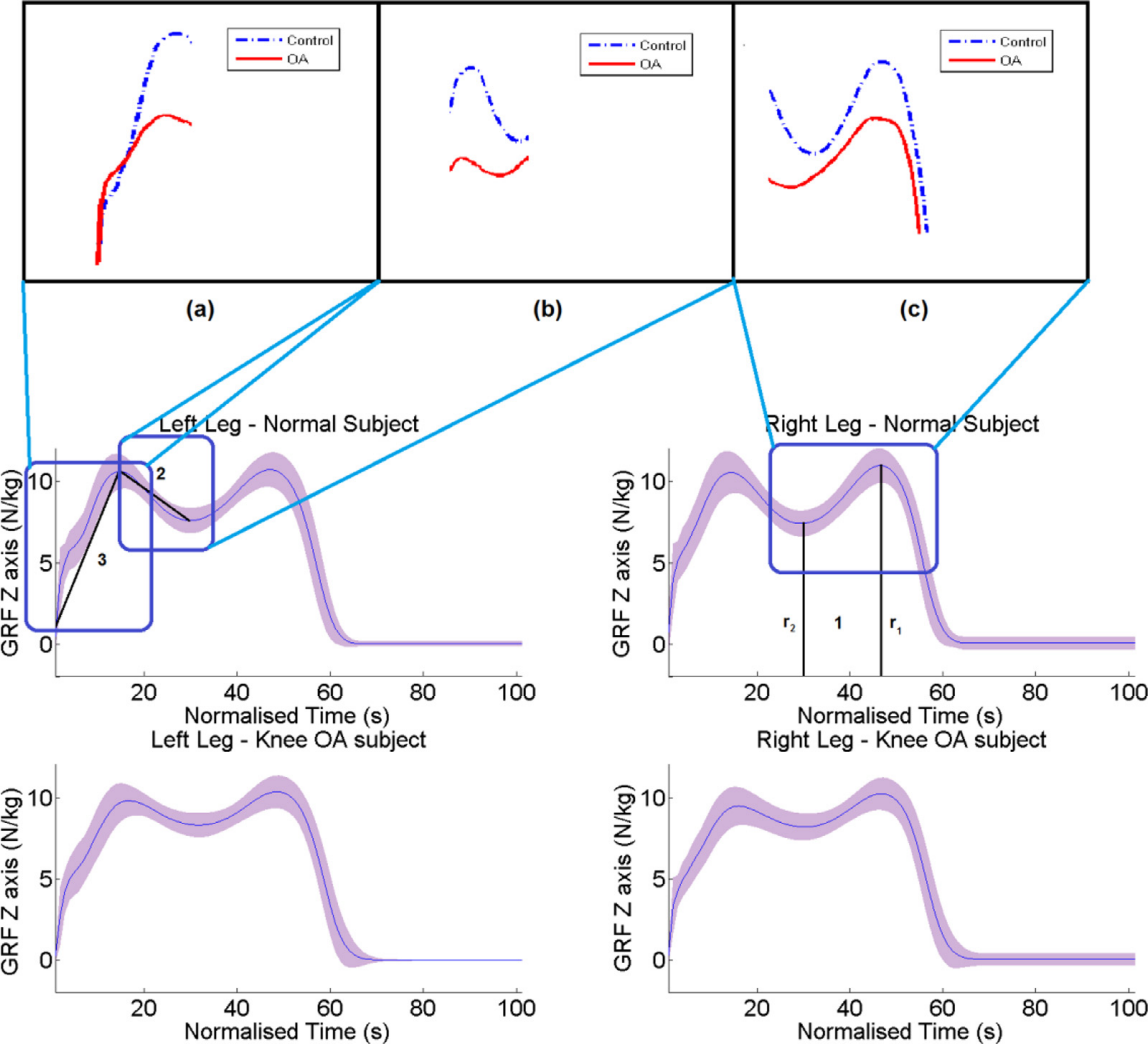
GRF-Z for control subjects and knee OA subjects per leg. The blue curve corresponds to the mean GRF-Z curve, whereas the purple shaded region indicates the precision of plus minus one standard deviation. The most discriminative parameter, indicated by 1 is a measure of the M-shape of the GRF-Z. The second parameter is the slope noted by 2 and the third parameter is the slope indicated by 3. Subject specific examples for each of the discriminating parameter are zoomed in the upper part of the figure. The blue curve is produced by a subject with no OA, whereas the blue one by a subject that suffers knee OA.

With respect to GRF-Z the 3 parameters that bear the most discriminating power are (Fig. 4): (1) the ratio of the peak push off value over the minimum value during mid-stance. As it is demonstrated in Fig. 4 subjects that suffer from knee OA have a tendency to apply less force during mid-stance. (2) The slope defined from the first peak to the minimum value between the first and second peak (Fig. 4(b)) that is related to the reduction on the GRF-Z due to knee flexion. (3) The slope defined from the beginning of the gait cycle to the first peak, as depicted in Fig. 4(a) that is related to weight acceptance. This means that OA subjects have flatter GRF patterns when compared to normal subjects and that knee OA subjects have a more gradual weight acceptance.

**Fig. 5:**
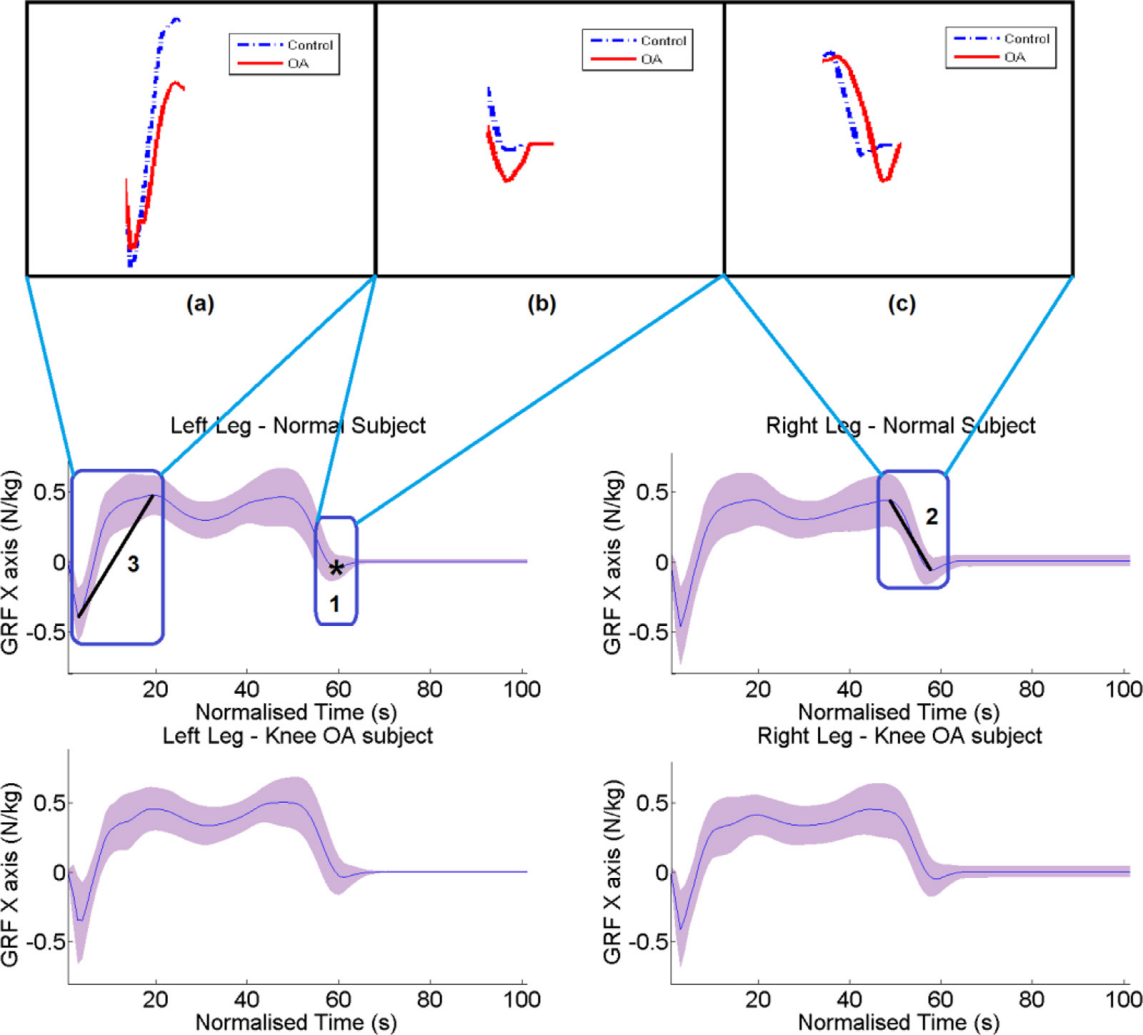
GRF-X for control subjects and knee OA subjects per leg. The blue curve corresponds to the mean GRF-X curve, whereas the purple shaded region indicates the precision of plus minus one standard deviation. The minimum value obtained before the end of the stance phase for the left leg, denoted by ‘*’, as well as by “1” is the most discriminative parameter. The second more discriminative parameter is the slope denoted with 2, and accordingly 3 indicates the slope that is ranked third with respect to discriminative power. Subject specific examples for each of the discriminating parameter are zoomed in the upper part of the figure. The blue curve is produced by a subject with no OA, whereas the blue one by a subject that suffers knee OA.

For the GRF-X axis the most important parameters are (Fig. 5): (1) the minimum value obtained before the end of the stance phase for the left leg, as depicted in Fig. 5(b) that is related to the medio-lateral force at toe off. (2) The slope between the second peak and the toe off of the right leg (Fig. 5(c)), that is related to moving medially from the peak lateral force. (3) The slope defined from the first minimum value of the gait cycle to the first peak (Fig. 5(a)), that is related to development of the lateral force during weight acceptance.

**Fig. 6:**
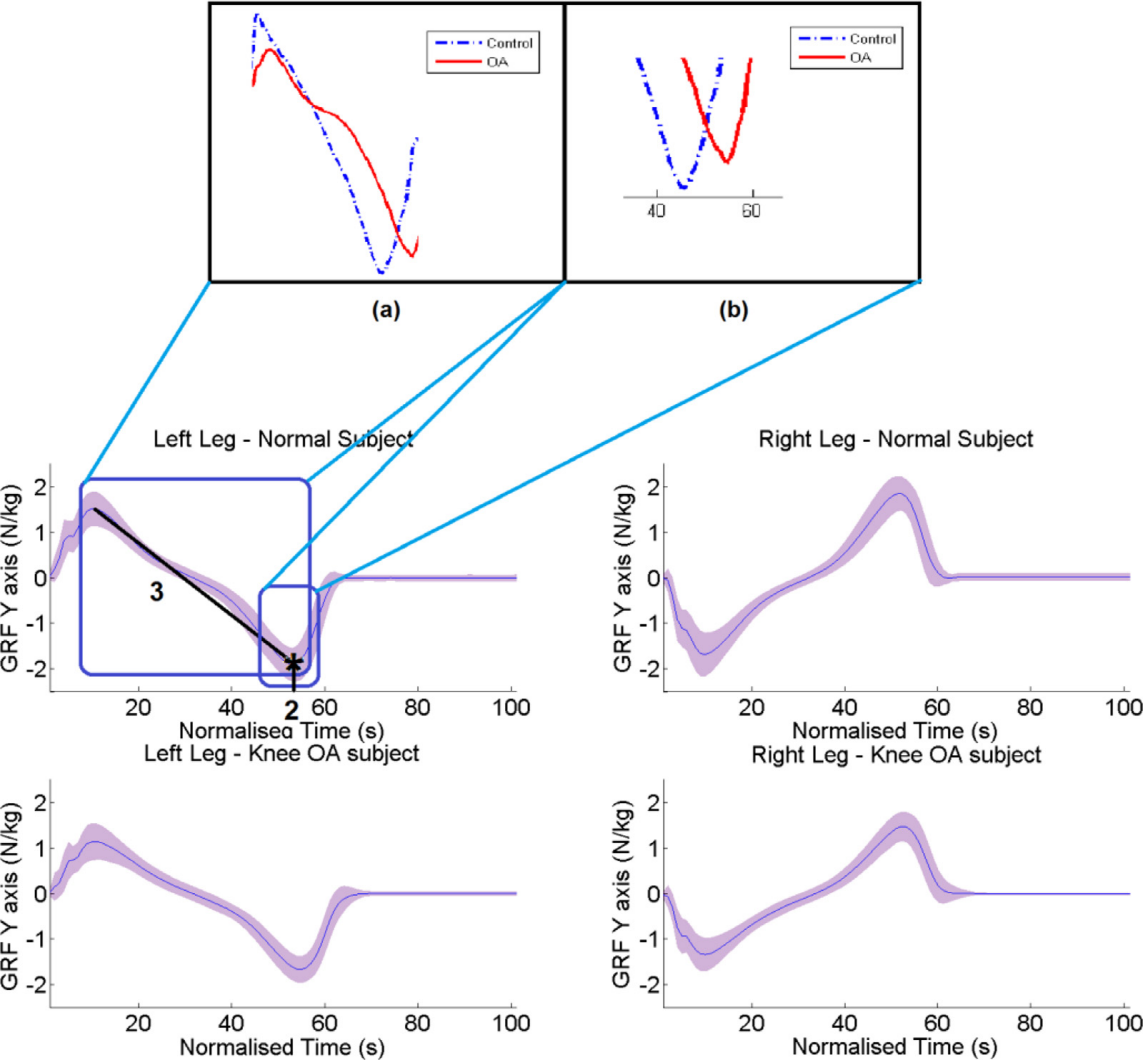
GRF-Y for control subjects and knee OA subjects per leg. The blue curve corresponds to the mean GRF-Y curve, whereas the purple shaded region indicates the precision of plus minus one standard deviation. The first most informative parameter is the minimum value during pre-swing indicated by 1. The slope stated by 2 is the second most informative parameter, whereas the third one is the total standard deviation of the GFRZ-Y curve. Subject specific examples for each of the discriminating parameter are zoomed in the upper part of the figure. The blue curve is produced by a subject with no OA, whereas the blue one by a subject that suffers knee OA.

For the GRF-Y axis the most important parameters are (Fig. 6): (1) the difference in standard deviation between the two legs of anterior–posterior force. (2) The time stamp of the minimum value of the left leg, as shown in Fig. 6(b), that is the time of the peak push off in posterior direction. (3) The slope from the maximum value to the minimum value for the left leg, as demonstrated in Fig. 6(a), that is the shear force moving from the peak anterior breaking force to the peak posterior push off force.

We can consider that the proposed approach classifies a subject correctly if: (1) the subject declares that he/she has no OA and the proposed approach output≤0.5 or (2) the subject suffers from knee OA and the proposed approach output > 0.5. In any other case a misclassification occurs. The results for this protocol are depicted in Table 3(a) for GRF-Z, Table 3(b) for GRF-X, and Table 3(a) for GRF-Y. Table 3(d) refers to the linear combination per subject for all GRFs, i.e. the final regression value for each subject is the mean of the regression values calculated for GRFZ, GRFX, and GRFY. Additional figures of merit are calculated for the confusion matrixes presented in Table 3. Those comprise sensitivity, specificity, accuracy and F1 score and are demonstrated in Table 4.

With respect to regression accuracy the mean squared error for the GRF-Z is 0.64, for the GRF-X it is 0.67, and for GRF-Y it is 0.64. If we combine the three axes in a linear manner, i.e. if we consider as final regression value per subject the mean value over all three axes, then the mean squared error drops to 0.59. It is noted that the regression values are averaged across trials for the same subject due to the subject-independent protocol.

Also, to prove the stability, robustness, and generalisation ability of the proposed method, a 5-fold cross validation is performed. Once again, the subjects for the 5 different training/testing splits are selected in a subject-independent manner. In the cross-validated case, the combined over the three axes mean squared error is 0.44 ± 0.09, whereas the mean accuracy equals 72.61% ± 4.24%.

To overcome the limitations that bilateral knee OA subjects are introducing, an alternative configuration of the dataset is tested. In this case, all subjects with OA in both knees, along with their age and gender matched were removed. This leaves us with 36 subjects that exhibit OA in one knee along with their 36 respective age and gender matched subjects. The rest of the computer system configuration remains the same. The results for this protocol are depicted in Table 5(a) for the linear combination per subject for all GRFs, whereas the figures of merit are demonstrated in Table 5(b). To comment on those results, accuracy for all GRFs has risen from 65.22% to 77.78%. This can be attributed to the fact that the exclusion of the subjects that have OA in both knees leads to a more homogeneous dataset, so the discrimination between the two categories is more consistent.

To compare the trials, for the case of unilateral knee OA and their controls, we calculated the frequency of subjects that (i) had 3 trials classified correctly, (ii) had 2 trials classified correctly, (iii) had 1 trial classified correctly, and (iv) had no trial classified correctly. 22 subjects (or 61.1%) belong to the first category; 6 subjects (or 16.7 %) belong to the second category; 4 subjects (or 11.1%) belong to the third category; and 4 subjects (or 11.1%) belong to the third category.

## 4 Discussion

This paper presents a novel computer system that automatically (i) estimates the degree of knee OA based on GRFs; (ii) discriminates the most important parameters for reaching its decision; those parameters are in fact in line with the literature, as detailed later on this Section and (iii) produces a set of rules, presented in this paper as binary decision trees, that can be alternatively seen as a set of if-then-else arguments; these rules we propose are easy from a clinical perspective.

Additional experimental results demonstrating the effect of thresholding as well as using alternative machine learning techniques, namely support vector machines, as well as additional training/testing splits, namely leave-one-subject-out, are presented in Supplementary Material.

The presented protocol leads to a high number of false negatives. Approximately 20% of subjects that claimed they did not have knee OA, presented with gait patterns similar to those of subjects that suffer knee ΟΑ. This may potentially be attributed to the fact that our healthy population were not investigated for joint abnormalities using imaging. As such, they may had early unknown signs of knee joint changes that led them to work with a gait pattern that bears some resemblance with that seen in people with knee OA. However, this is a speculation and would require further research to validate. Our findings clearly indicate that for verification an imaging assessment of the healthy subjects is required. Radiographic assessment of the healthy subjects is part of our proposed future work.

Our method has its own limitations. First of all, the method is not validated against radiographic imaging, such as X-rays or MRIs which often are used for OA diagnosis. However, using figures in the scientific literature indicates that less than 50% of people with evidence of OA on plain radiographs have symptoms related to these findings as proved by Hannan et al. [33]. Therefore, the ‘clinical endpoint’ is more difficult to establish as explained by Hunter et al*.* [34]. To conclude, the work of Zhang et al*.* [35] proves that there is no gold standard in the diagnosis of knee OA. However the knee OA subjects were identified by experienced orthopaedic clinicians and GPs based on their clinical examination findings and medical records. A fraction of knee OA subjects had been referred from their GP for an X-ray or MRI (however, these images were not always available and any grading of OA severity is dependent of the expertise of their clinician). Healthy volunteers were assessed for any exclusions criteria such as knee pain or limitation in functional ability, but did not have this confirmed through imaging; as such they may have had early signs of OA that were undetected. However, this study aims to work as a proof of concept, rather than a validation study. The next step is to obtain ethics and funding to recruit a larger number of subjects all of which will undergo MRI at the respective hospital department at the time of data collection. This will allow us to confirm the presence or absence of imaging signs of knee OA. Also, the results although clinically relevant cannot be used in the everyday clinical practice without further work including validating the suitability of the selected features as knee OA markers and, ultimately, risk factors.

On the advantage side, the parameters that we discriminate as most informative in this study are in line with the findings in the related literature. OA subjects are thought to adopt gait compensation strategies to reduce pain or the moments generated about the knee. Such strategies may provide insight into the altered parameters noted here. For example, reduced gait speeds may be adopted by patients in order to reduce medial compartment loading in OA subjects, as suggested by Mündermann et al. [20], through reduction of GRF-Z peak amplitude and loading rate as demonstrated by Zeni and Higginson [21]. Reduced knee excursion in the sagittal plane during the stance phase of gait has been reported in knee OA subjects by Childs et al*.* [22] as well as by Schmitt and Rudolph [23] and related to weakness of the quadriceps muscle; this would also affect the rate of force development in GRF-Z. Other strategies are thought to alter medio-lateral knee loading according to Simic et al*.* [24], including increased varus thrust as proposed by Chang et al*.* [25] and lateral trunk lean, which is thought to change the location of the centre of the mass in the frontal plane as explained by Mündermann et al*.* [26] and Hunt et al*.* [27] and would therefore alter GRF-X. Increased trunk lean was also associated with pain in OA subjects as shown by Bechard et al*.* [28]. Finally, alterations in foot (toe-out) angle, are postulated to mediate medio-lateral knee forces and pain as suggested by Bechard et al. [28], Lynn and Costigan [29], and Simic et al. [30] and would alter shear forces both in the medio-lateral and antero-posterior directions.

Comparing the work shown here with the previous research presented by Kotti et al*.* in [31], the main difference lies on the research focus and methodology. The work of Kotti et al*.* [31] focused on understanding the motor behaviour by deconstructing its complexity. In more detail, it was studied how to deconstruct GRFs into a low-dimensional space and if this deconstruction of GRFs was capable of discriminating between subjects with and without knee OA. Considering the methodology, probabilistic principal component analysis (PPCA) was used for dimensionality reduction and the classification was done by means of a Bayes classifier. All the axes were considered concurrently, that is no results were available per axis, and no feature engineering took place. The use of PPCA means that a direct physical interpretation of the results was not possible. Moreover, the approach presented by Kotti et al*.* [31] was not designed exclusively for GRFs and could be transferred to other signals, such as EMGs, since no feature engineering is required. On the common methodology side, both works are subject-independent and use a cross-validated protocol.

The advantages of our method compared with the related research summarized in the Introduction Section that also uses GRFs (that is Moustakidis et al*.* [10] and Mezghani et al*.* [11]), are that (i) a greater number of subjects is exploited; (ii) the experimental protocol is subject-independent; and (iii) the experimental protocol is 50% training/50% testing. However, this has an effect on the accuracy of the results presented here. For example, Moustakidis et al*.* [10] report an accuracy of 93.4%, using a subject-dependent 10-fold cross validation protocol over 214 trials of just 36 subjects. Accuracy is boosted since the experimental protocol is both subject-dependent and 90% training/10% testing, thus less challenging than the subject-independent 50% training/50% testing exploited in this work and also due to the feature engineering, rendering the features not directly clinically interpretable. Also, the 2 force plates used by Moustakidis et al*.* [10] are embedded into a treadmill, rather than in a walkway, as in the presented approach. There is an argument in the research community whether treadmill gait data are different from overground walking gait according to Warabi et al*.* [32]. Referring to the system presented by Mezghani et al*.* [11], the experimental protocol in this case is leave-one-subject out, so subject independent, but still less challenging than the leave-half-the-subjects-out tested here. The number of subjects is 42, so less than half of those tested for this paper. Moreover, we consider all the trials provided by each subject, rather than averaging across trials in order to calculate the mean GRFs, as is the case of Mezghani et al*.* [11]. Averaging disregards the intra-subject variability, rendering the problem less complex. One of the main advantages of our approach is that it simultaneously discriminates between subjects that have knee OA by extracting the most informative parameters. Our aim is to create a clinically relevant tool that enables the physician to see the influence of each parameter upon discrimination, as suggested by Beynon et al*.* [8]. Also, in both cases we need to identify whether the proposed tool makes decisions in line with clinical opinion. Additionally, our study has a common point with that of Moustakidis et al*.* [10], since they both decompose the complex knee OA problem into simpler binary sub-problems via tree structures. However, for the random forest approach, its robustness is mathematically proven, it is robust to overfitting, and it does not utilise heuristics that are subjectively defined. An additional advantage of this study is that since we do not transform our initial parameters we do not need to map them back to the original space, where they have a physical meaning. Such a mapping is subjective and may lead to ambiguities. For example, the parameters derived by Deluzio and Astephen in [9], namely the knee flexion moments during stance, knee adduction moments during the stance phase, and knee flexion ranges of motion throughout the gait cycle are qualitative observations. In our work the parameters are strictly, quantitatively defined. The same argument applies to discrete wavelet decomposition, where a mother wavelet Symlet is utilised to capture the temporal information in the work of Mezghani et al. [11]. However, it is unclear which temporal information was retained and why. Finally, this study takes extra care to use a subject-independent protocol to boost generalization. Subject dependent protocols can lead to systems of higher accuracy, since a subject already seen during training is re-tested during the testing phase, as done by Beynon et al*.* [8]. However, such systems may not be robust when they actually see a subject outside of the training population.

## 5 Conclusion

To conclude this paper has proved the suitability of random forests for analysing ground reaction forces in order to distinguish knee OA patients from healthy ones. Moreover, it has managed to provide a set of 9 features, 3 per axis, that are more discriminative of knee OA. The suitability of those features has been verified by the related bibliography. However, our method manages to combine those features in a rule-based way, instead of using them independently. Moreover, the rule-based core of the proposed system is close to the clinical rationale. To boost intra-subject consistency subjects were asked to walk along the walkway 3 times. Mean squared error is 0.44 ± 0.09, whereas the mean accuracy equals 72.61% ± 4.24% in a subject-independent protocol. However, further studies are needed to validate those findings as well as to collect data whose ground truth is derived through imaging. Our ultimate clinical vision is to create an objective, sensitive, diagnostic tool and to personalise health care, since each individual patient traverses the regression trees in a unique way.

## Conflict of interest

None declared

## Ethical approval

Ethical approval for this study was obtained from the South West London Research Ethics Committee and written informed consent was obtained from all participants.

## Figures and Tables

**Table 1:** Mean value and standard deviation about the age, height, weight, and sex of the control and the knee OA subjects.

	Controls	Knee OA
	(47 subjects)	(47 subjects)
Age (years)	54.4 (13.3)	58.1 (12.7)
Height (mm)	1705.7 (88.9)	1695.8 (113.2)
Weight (kg)	69.4 (10.6)	76.2 (14.4)
Male/Female	22/25	22/25

**Table 2:** Basic statistics and signal processing features that are computed for all three axes. Additionally, axis-specific parameters are computed.

GRZ-Z	GRF-X	GRF-Y
Maximum, mean, median, and standard deviation and the differences between the aforementioned values for the both legs.		
Skewness, kurtosis, interquartile range, 75th percentile, and 90th percentile.		
Energy and the power spectral density of each leg.		
The length of the stance phase, along with the Spearman correlation between the two legs.		

**Table 3:** Confusion matrixes using the 0.5 knee OA threshold when exploiting (a) GRF-Z, (b) GRF-X, (c) GRF-Y, and (d) combined for all three axes. In case a subject has more than one trial available, the final regression value is calculated by averaging over the trials.

**Table 4:** Figures of merit, namely sensitivity, specificity, accuracy, and F1 score for the confusion matrixes exhibited in Table 3, specifically for (a) GRF-Z, (b) GRF-X, (c) GRF-Y, and (d) combined for all three axes.

**Table 5:** (a) Confusion matrix for the case of unilateral knee OA subjects. In this case subjects exhibiting OA in both knees, along with their age and gender matched ones have been excluded. The rest of the computer system configuration remains the same. (b) Figures of merit for the confusion matrix that appears in Table 5(a).

## Appendix Supplementary materials

application 1 Image, application 1
