# Supplementary material for: Detecting knee osteoarthritis and its discriminating parameters using random forests
Source: Med Eng Phys. 2017 May;43:19–29. doi: 10.1016/j.medengphy.2017.02.004 (PMC5390773; doi:10.1016/j.medengphy.2017.02.004)
Supplement: application 1 [file mmc1.doc]

**Supplementary Material**

Additional trees for GRF-X and GRF-Y. Each tree is one of the ten that comprise the random forest.


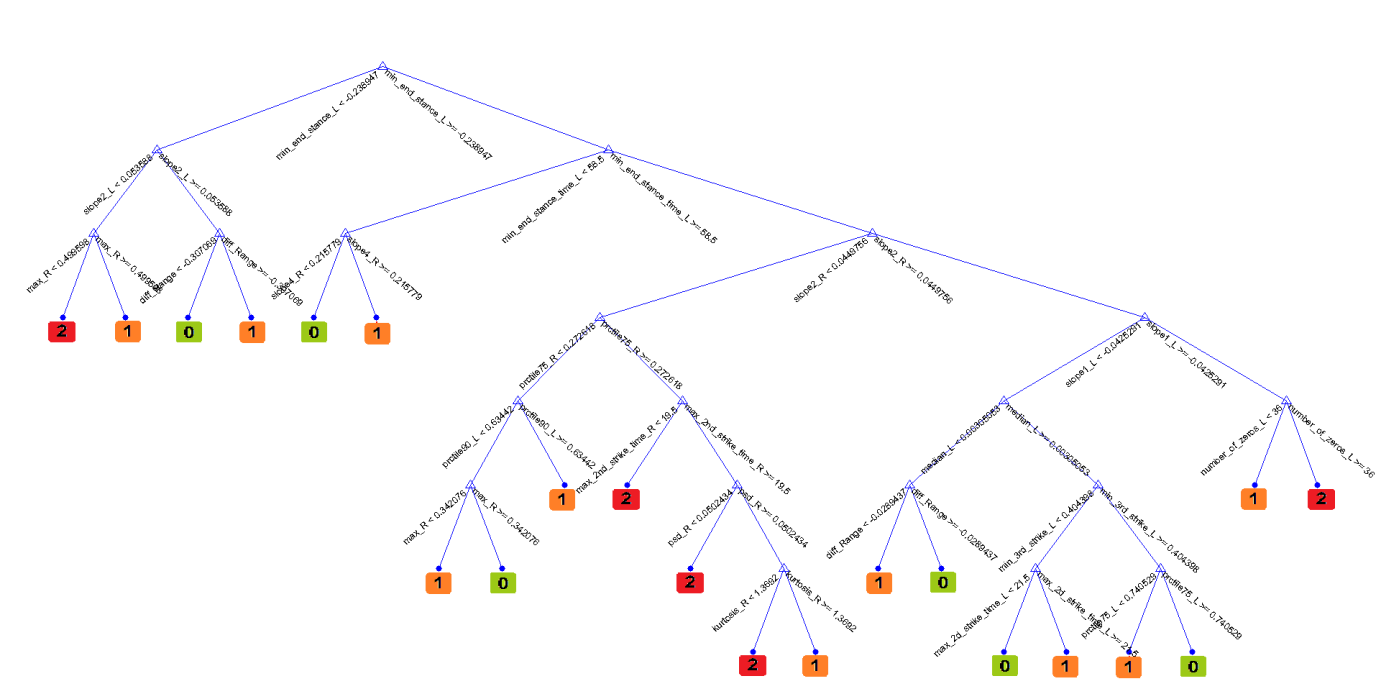


Figure S1: One of the regression trees comprising the random forest for the GRF over X-axis. The regression tree is built using a random subset of the parameters extracted for GRF-X.


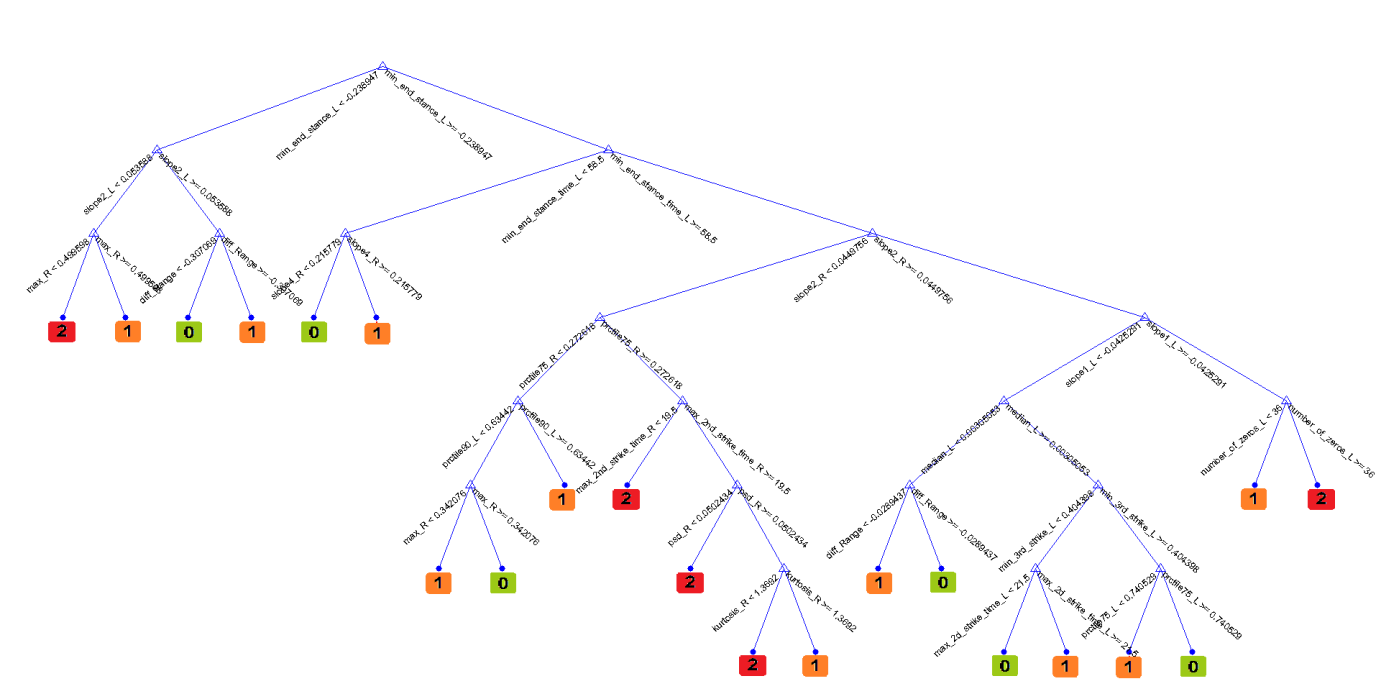


Figure S2: One of the regression trees comprising the random forest for the GRF over Y-axis. The regression tree is built using a random subset of the parameters extracted for GRF-Y.

Additional experimental results

To provide a better insight to the threshold effect on the proposed method’s efficiency, Figure S3 exhibits the results using different thresholds. Specifically, in Figure S3(a) the accuracy against several threshold values is depicted; the ROC curve is shown in Figure S3(b); whereas sensitivity and specificity for several threshold values is drawn in Figures S3(c) and S3(d), respectively.


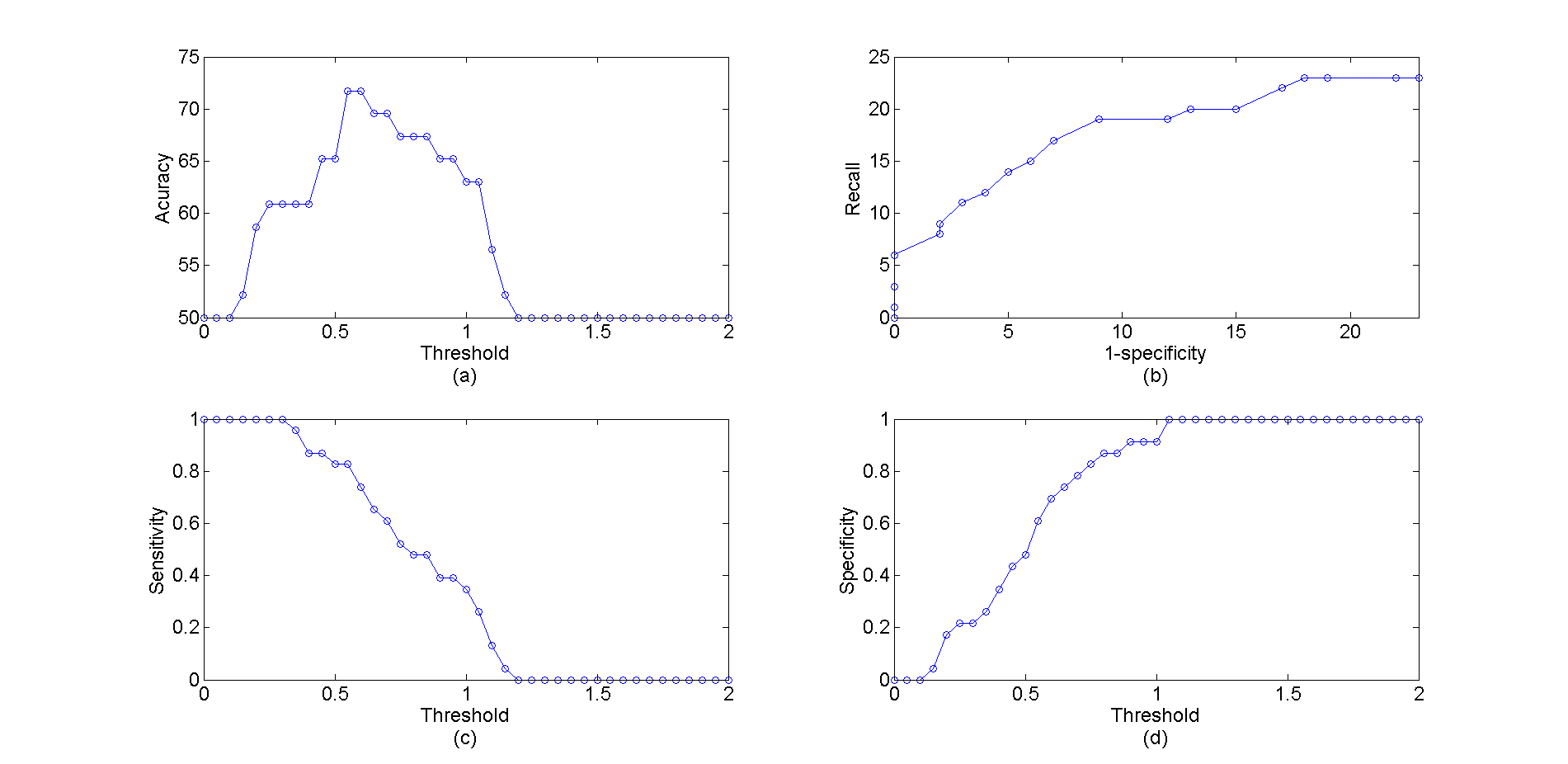


Figure S3: The behaviour of the algorithm for several threshold values in the range 0-2. Subfigure 7 (a) refers to the accuracy, (b) is the ROC curve, (c) the sensitivity and (d) the specificity. The best accuracy is 71.74% and corresponds to a threshold value of 0.55.

To facilitate comparison with other approaches, the same experiment was repeated by substituting the random forest with a linear SVM regressor. SVM avoids overfitting by finding the maximum-margin hyperplane, so it exhibits good generalization performance and is a common choice for a testbed. The rest of the experimental protocol remains the same. When employing an SVM, the combined over the three axes mean squared error is 0.63, whereas the confusion matrix for the linear combination per subject for all GRFs is depicted in Table S1. For Table S1 sensitivity is 73.91%, specificity is 52.17%, accuracy is 63.04% and F1 score is 66.67%. SVM accuracy equals 63.04% and is smaller than the random forest accuracy that is 65.22%.

| GRFZ, GRFX, GRFY  SVM | | **Estimated by the regression forest** | |
| --- | --- | --- | --- |
|  |  | No OA | Has OA |
| **True value** | No OA | 12 | 11 |
|  | Has OA | 6 | 17 |

Table S1: Confusion matrixes using the 0.5 knee OA threshold when combined for all three axes. In case a subject has more than one trial available, the final regression value is calculated by averaging over the trials.

Finally, an additional experimental configuration using the random forest was tested. In this case, a leave-one-subject-out protocol was adopted. In that case, one subject is used for testing and the remaining 93 for training. The rest of the experimental configuration is retained the same as before. Here, the mean squared error for all 3 axes is 0.10, whereas the accuracy equals 77.42%. The corresponding confusion matrix can be seen in Table S2. For Table S2 sensitivity is 80.43%, specificity is 74.47%, and F1 score is 77.89%.

| | GRFZ, GRFX, GRFY  Leave-one-subject-out | | **Estimated by the regression forest** | | | --- | --- | --- | --- | | No OA | Has OA | | **True value** | No OA | 35 | 12 | | Has OA | 9 | 37 | |  |
| --- | --- | --- | --- | --- | --- | --- | --- | --- | --- | --- | --- | --- | --- | --- |

Table S2: Confusion matrixes using the 0.5 knee OA threshold for all three axes for the case of leave-one –subject-out.
